# LINC00941 promotes oral squamous cell carcinoma progression via activating CAPRIN2 and canonical WNT/β‐catenin signaling pathway

**DOI:** 10.1111/jcmm.15667

**Published:** 2020-07-21

**Authors:** Yilong Ai, Siyuan Wu, Chen Zou, Haigang Wei

**Affiliations:** ^1^ Foshan Stomatological Hospital School of Stomatology and Medicine Foshan University Foshan, Guangdong China

**Keywords:** CAPRIN2, LINC00941, long non‐coding RNA, Oral Squamous Cell Carcinoma (OSCC), WNT signaling pathway

## Abstract

Dysregulation of long non‐coding RNAs (lncRNAs) has been implicated in many cancer developments. Previous studies showed that lncRNA LINC00941 was aberrantly expressed in oral squamous cell carcinoma (OSCC). However, its role in OSCC development remains elusive. In this study, we demonstrated that in OSCC cells, EP300 activates LINC00941 transcription through up‐regulating its promoter H3K27ac modification. Up‐regulated LINC00941 in turn activates CAPRIN2 expression by looping to CAPRIN2 promoter. Functional assays suggest that both LINC00941 and CAPRIN2 play pivotal roles in promoting OSCC cell proliferation and colony formation. In vivo assay further confirmed the role of LINC00941 in promoting OSCC cell tumour formation. Lastly, we showed that the role of LINC00941 and CAPRIN2 in OSCC progression was mediated through activating the canonical WNT/β‐catenin signaling pathway. Thus, LINC00941/CAPRIN2/ WNT/β‐catenin signaling pathway provides new therapeutic targets for OSCC treatment.

## 1 INTRODUCTION

Oral cancer poses a great threat to human health. Based on the histopathological classification, 90% of oral cancers belong to oral squamous cell carcinoma (OSCC).^1^ Annually, over 300 000 new patients were diagnosed with oral cancer and 145 000 deaths were linked to this disease worldwide.^2^ Smoking, excessive consumption of alcohol and other factors including HPV infection have been recognized as high‐risk factors for oral cancer development.^3^, ^4^ The combination uses of chemotherapy, surgery and radiotherapy remain the mainstay for oral cancer treatment.^5^, ^6^ These treatments are efficient for early‐stage oral cancer patients, but are not ideal for those relapsed or late‐stage patients.^7^ Even though molecular targeted therapies like inhibitors or monoclonal antibodies against EGFR have been used in the clinical,^8^, ^9^ drug resistance during treatment greatly limits their therapeutic efficacy. Thus, identifying new molecular targets are needed for improving oral cancer treatment.

The advancement of whole‐genome and whole‐exome sequencing greatly accelerated the studies on DNA regions that do not encode proteins. Within the genome, only 3% of DNA encodes protein, those non‐coding regions are used to be regarded as ‘junk DNA’.^10^ However, recent exome sequencing discovered that 66% of RNA transcripts came from non‐coding regions. These non‐coding RNAs include long non‐coding RNA (lncRNA), microRNA (microRNA) and circular RNA.^11^, ^12^ LncRNAs are a group of RNAs that have limited or no protein‐coding potency. LncRNAs are synthesized by RNA polymerase II with length typically longer than 200bp. Although lncRNAs do not encode proteins, they share many biological characteristics with mRNAs.^13^, ^14^ Recent evidence suggests that lncRNAs play pivotal roles in many biological processes, such as inflammation, cell growth, cell differentiation and tumorigenesis.^13^, ^14^ Dysregulation of lncRNAs has been implicated in oral cancer development. For example, MALAT1, a highly conserved lncRNA, was discovered highly expressed in oral squamous cell carcinoma (OSCC). Silencing MALAT1 greatly impaired epithelial‐mesenchymal transition‐mediated cell migration and invasion through suppressing N‐cadherin and Vimentin expression.^15^ Increased HOTAIR expression is associated with OSCC cell stemness, invasion and metastasis.^16^, ^17^ LncHIFCAR was reported to act as a HIF‐1a co‐activator that drives oral cancer progression.^18^

WNT signaling pathway begins from WNT‐protein ligand that passes signals into cell through binding to cell surface receptors.^19^ Based on different molecules involved in signaling transduction, WNT signaling pathway can be divided into three different pathways, including the canonical WNT pathway, the non‐canonical planar cell polarity pathway and the non‐canonical WNT/calcium pathway. All of these three pathways are activated upon WNT‐protein ligand binding to frizzled family receptor. The roles of WNT signaling pathway have been extensively studied in carcinogenesis and embryo development. Dysregulation of WNT signaling pathway has been implicated in many cancers including breast cancer, prostate cancer, liver cancer and others.^20^, ^21^, ^22^

By comparison of RNA‐seq data sets obtained from primary head and neck squamous cell carcinoma (HNSCC) tumour and normal tissues, Koyo et al identified 15 lncRNAs including LINC00941 that are aberrantly expressed in tumour tissues.^23^ However, the role of LINC00941 in OSCC remains unknown. In this study, for the first time, we demonstrated that elevated LINC00941 promotes OSCC progression through up‐regulating CAPRIN2 expression, which further activates canonical WNT/β‐catenin signaling pathway. Thus, LINC00941 could be a useful new therapeutic target in OSCC.

## 2 MATERIAL AND METHODS

### 2.1 Cell lines and patient tissues

OSCC cell lines used in this study, including HSC‐3, SCC‐9, CAL‐27 and OSC‐19, were purchased from National Infrastructure of Cell Line Resource, Shanghai, China. Cells were cultured in Dulbecco's modified Eagle's medium (DMEM) supplemented with 10% foetal bovine serum (FBS). Normal oral keratinocytes HOK was purchased from Creative Bioarray, USA. Cells were cultured in human oral keratinocytes. Normal tongue tissues were collected from patients undergoing surgery in Foshan Stomatology Hospital. Twelve paired snap‐frozen primary OSCC tumour tissues and its adjacent normal non‐cancerous mucosa tissues were obtained from Foshan Stomatology Hospital. Written informed consents were obtained from all participants.

### 2.2 Cell transfection

Cells were digested with 0.25% trypsin and seeded into 6‐well plates 1 day earlier before transfection. Transfection was done with Lipofectamine 2000 (Life Technologies) according to the manufacturer's instructions. The fresh medium was changed 6 hours after transfection. Experiments were done 24 or 48 hours later.

### 2.3 Quantitative reverse transcription PCR (RT‐qPCR)

Transcription level was determined by RT‐qPCR. Briefly, cells were harvested and washed with PBS once. RNA was extracted with an RNA extraction kit purchased from BioChain (Cat: K1341050). One ug RNA was reverse‐transcripted into cDNA and used as template for gene quantification. The relative transcription level was calculated with 2^‐^∆∆^ct^_._

### 2.4 Luciferase assay

LINC00941 promoter ~1000bp upstream of transcription start site (TSS) was PCR amplified and ligated into pGL3‐basic vector through infusion clone. pGL3‐LINC00941 was transfected into cells with Lipofectamine 2000. Cells were treated with different doses of EP300 inhibitor GNE‐049 6 hours after transfection. The activity of luciferase was tested with Dual‐Luciferase reporter assay kit (Promega Corporation). Renilla was used to normalize luciferase activity among different groups.

### 2.5 Northern blot

Northern blot was used to detect LINC00941 transcripts in OSCC and HOK cells. Briefly, cells were harvested and washed with ice‐cold PBS. RNA was extracted with RNA extraction kit. The same amount RNA was loaded into 0.8% denaturing agarose gel. RNA was separated by electrophoresis and was transferred to a membrane. DNA probe against LINC00941 was labelled with ^32^P and was used to detect LINC00941 expression.

### 2.6 Cell proliferation

Twenty‐four hours after transfection, cells were digested and seeded into 24‐well plate at a concentration of ~0.05 million/mL. Cells were then allowed to outgrowth and collected at days indicated. The cell number was counted with a hemocytometer. At least three replicates were performed at each time‐point.

### 2.7 Cell colony formation

Cell colony formation indirectly reflects the tumour formation ability in vivo. OSCC cells were transfected with plasmids as indicated. Twenty‐four hours after transfection, cells were collected and counted. ~600 cells were seeded into 6‐well plates. After ~14 days, when colonies were visibly observed, medium was removed, and colonies were washed with ice‐cold PBS twice. Five ml ice‐cold 100% methanol was added into plates for colony fixation. Colonies were then stained with 0.1% crystal violet. The background was destained by washing plates multiple times with flowing water until the colony‐free area turned into white. Colonies were imaged with HP scanner, and colony numbers were counted and statistically analysed.

### 2.8 Cell cycle

Propidium iodide (PI) was used to stain cells for cell cycle detection. Briefly, after transfecting cells for 48 hours, cells were harvested and fixed with 90% ice‐cold ethanol at −20°C overnight. The next day, cells were allowed to equilibrate to RT by incubating at RT for 5 minutes. Cells were then washed with PBS at RT twice and stained with 1 mL PI staining solution (50 µg/mL; 1 mg/mL of RNase A, 0.1% Triton X‐100 in PBS) by incubating at RT for 30 minutes in the dark. Flow cytometry was applied to detect the cell cycle.

### 2.9 CRISPR‐mediated gene knockout and inactivation

pLentiCRISPR v2 containing sgRNAs targeting EP300 and CAPRIN2 were purchased from GenScript, USA. One ug sgRNA vectors, 500 ng VSVG and 800 ng PsPAX2 were cotransfected into 293T cells by using Lipofectamine 2000. After incubation for 6 hours, fresh medium was changed. Lentiviruses were collected 48 hours later and were used to infect target cells. The efficiency of gene knockout was validated by Western blots.

To silence LINC00941 expression, OSCC cell lines stably expressing dcas9‐KRAB‐MeCP2 were first constructed by packaging dcas9‐KRAB‐MeCP2 into lentiviruses and used to infect OSCC cells. Infected cells were then selected with 5 ug/mL blasticidin for at least 5 days. The expression of dcas9 was validated by Western blots. LINC00941 TSS ‐500‐500bp region was used as a template to design sgRNAs. Designed sgRNAs were cloned into pLentiGuide‐puro vector and packaged into lentiviruses. OSCC cells stably expressing dcas9‐KRAB‐MeCP2 were infected with lentiviruses for 2 days and then selected with 3 ug/mL puromycin for another 3 days. The efficiency of CRISPRi was determined by RT‐qPCR.

### 2.10 Chromatin Immunoprecipitation quantitative PCR (ChIP‐qPCR)

Cells were harvested and crosslinked with 1% formaldehyde with shaking at RT for 10 minutes. Crosslink was then stopped by adding 20× 2.5 mol/L glycine (final concentration 125 nmol/L) followed by shaking at RT for 5 minutes. Cells were lysed with cell lysis buffer, and DNA was fragmented by sonication. After sonication, cell solution was centrifuged at maxi‐speed ~13000RPM for 10 minutes, and supernatants were collected and pre‐cleared with Protein A beads with shaking at 4°C for 30 minutes. Interested antibodies or IgG were added into cell solution to bind protein‐DNA complexes with shaking at 4°C overnight. The next day, Protein A beads were added into the cell solution to pull down antibody‐protein‐DNA complexes. Beads were then extensively washed, and DNA was eluted with DNA elution buffer. Proteinase K was used to reverse DNA‐protein complexes, and DNA was purified with a DNA purification kit (Qiagen). The amount of precipitated DNA was determined by qPCR.

### 2.11 Quantitative analysis of chromosome conformation capture assays (3C‐qPCR)

3C‐qPCR was performed based on published protocol.^24^ Briefly, cells were fixed with 1% formaldehyde and DNA was digested with XbaI (NEB) with shaking at 4°C overnight. DNA was diluted with DNA dilution buffer, and DNA ends were ligated with T4 DNA ligase. DNA was then purified, and DNA‐DNA interaction was quantified by qPCR.

### 2.12 In vivo tumour formation

The animal experiment was strictly conducted based on the protocol of the institutional ethics board of the Foshan Stomatology Hospital of Foshan University. Every effort was made to minimize the pain of mice. Three‐week‐old mice purchased from Guangdong Medical Laboratory Animal Center (GDMLAC), China, were allowed to adaption in our facility for 1 week before performing any experiment. HSC‐3 cells stably expressing dcas9‐KRAB‐MeCP2 were transfected with scramble control or sgRNAs targeting LINC00941. After selection with puromycin for 3 days, cells were harvested, and 1 × 10^7^ cells were then subcutaneously injected into the right flanks of 4‐week‐old BALB/c nude mice. Mice conditions were monitored every day. Tumour volume was started to measure every week when tumour size reaching around 30 mm^3^. The experiment was stopped 4 weeks later. Mice were killed, and tumour weight was then measured.

### 2.13 Statistic

Data were analysed by Prism 7. The differences between untreated and treated groups were evaluated by unpaired t test. *P* < .05 was considered statistically significant.

## 3 RESULTS

### 3.1 LINC00941 is up‐regulated in OSCC cell lines and tumour tissues

**FIGURE 1:**
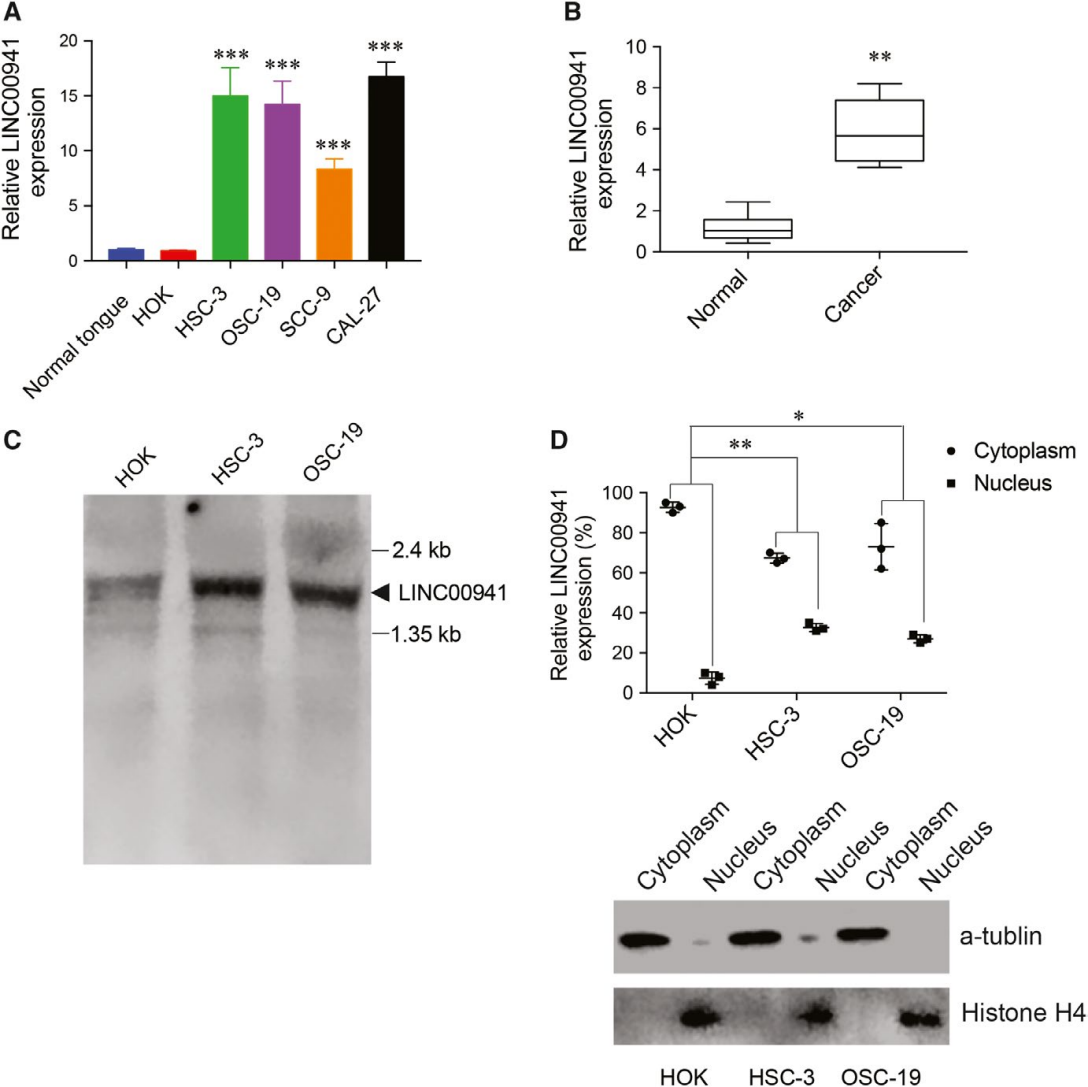
LINC00941 is up‐regulated in OSCC cell lines and tumour tissues. A, RT‐qPCR results of LINC00941 from OSCC cells, normal oral keratinocytes HOK and normal tongue tissue. B, RT‐qPCR results of LINC00941 between patient tumour tissues and normal tissues. C, Northern blot results of LINC00941 expression. Molecular weight was shown on right. D, Detection of LINC00941 distribution in cytoplasm and nucleus. a‐Tublin was used as a marker for cytoplasm, Histone H4 was used as a marker for nucleus. *P < .05, **P < .01, ***P < .001

Previous RNA‐seq analysis on 458 OSCC patient tumour tissues and 72 normal tissues discovered that long non‐coding RNA LINC00941 was highly expressed in tumours than normal tissues.^23^ To validate this, RT‐qPCR was first applied to determine LINC00941 expression in cells and tissues. Indeed, LINC00941 expression was highly up‐regulated in OSCC cell lines and OSCC tumour tissues when compared with normal cells and normal tissues, respectively (Figure 1A,B). To confirm this result, Northern blot was used to detect LINC00941 expression in HOK, HSC‐3 and OSC‐19 cells. As indicated, a clear band was observed between 1.35 and 2.4 kb, corresponding to the length of LINC00941 transcripts which is ~1.8 kb. Band intensity in HSC‐3 and OSC‐19 cells was also much higher than HOK cells (Figure 1C), suggesting the higher level of LINC00941 in OSCC cells than normal oral keratinocytes. The distribution of LINC00941 within the cell was also examined. Interestingly, although most of LINC00941 transcripts were located in the cytoplasm, the enrichment of LINC00941 in the nucleus was much higher in HSC‐3 and OSC‐19 cells than HOK cells (Figure 1D).

### 3.2 LINC00941 promoter H3K27 acetylation activates LINC00941 expression in OSCC cells

**FIGURE 2:**
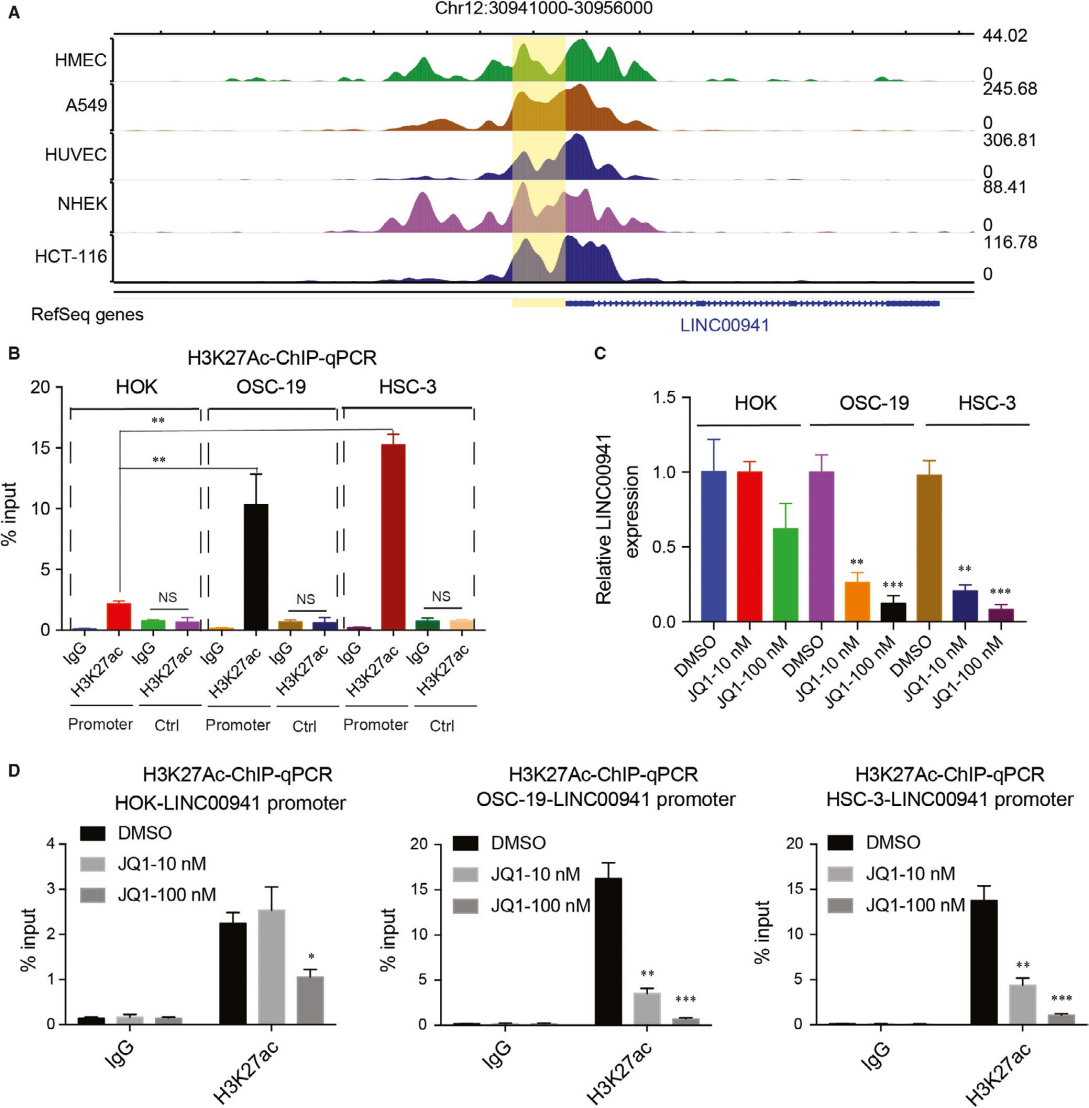
LINC00941 promoter H3K27 acetylation activates LINC00941 expression in OSCC cells. A, Diagram of H3K27ac signals on LINC00941 promoter across different cancer types. LINC00941 promoter was labelled with yellow. B, ChIP‐qPCR detects H3K27ac modification on LINC00941 promoter. DNA region adjacent to LINC00941 promoter without any H3K27ac signal was used as control. IgG was used as control. C, LINC00941 expression after treatment with different doses of JQ1 for 3 d. D, Detection of H3K27ac signals on LINC00941 promoter after treatment with different doses of JQ1 for 3 d. IgG was used as control. *P < .05, **P < .01, ***P < .001

**FIGURE 3:**
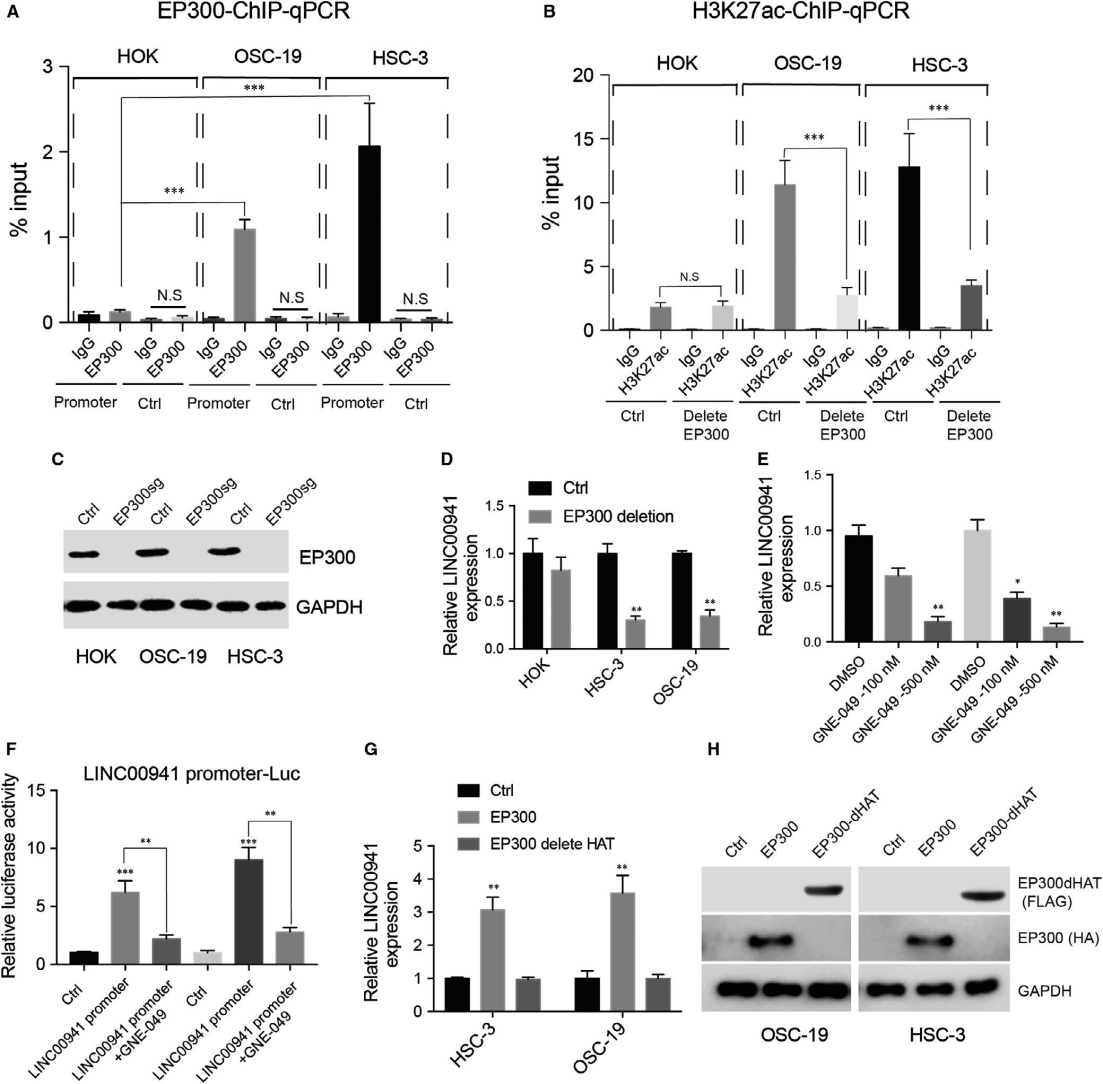
EP300 promotes LINC00941 promoter H3K27 acetylation and LINC00941 expression in OSCC cells. A, ChIP‐qPCR detects EP300 enrichment on LINC00941 promoter. DNA region adjacent to LINC00941 promoter was used as control. IgG was used as control. B, Detection of H3K27ac signals on LINC00941 promoter after depletion of EP300. sgRNA targeting non‐coding region was used as negative control, and IgG was used as control. C, Western blot results of EP300 after CRISPR‐CAS9‐mediated gene knockout, GAPDH was used as control. D, LINC00941 expression after deleting EP300. E, LINC00941 expression after treating HSC‐3 cells with different doses of EP300 inhibitor GNE‐049. F, LINC00941 promoter was cloned into pGL3‐basic vector and electroporated into HSC‐3 cells along with Renilla vector. Six hours later, cells were treated with 500 nmol/L GNE‐049 for 36 h. Luciferase activity was determined by dual‐luciferase assay. G, LINC00941 expression after exogenously introduce wild type or HAT domain deleted EP300. H, Western blot results of HA‐tagged EP300 and FLAG‐tagged EP300 in which HAT domain was deleted. GAPDH was used as control. *P < .05, **P < .01, ***P < .001

Histone H3K27 acetylation activates gene expression. To understand the mechanisms through which LINC00941 was activated in OSCC cells, H3K27ac ChIP‐seq data across different cancer cell lines were explored. As indicated, LINC00941 promoter region was greatly modified by H3K27ac among all the cells examined (Figure 2A), suggesting LINC00941 may be activated by its promoter H3K27ac modification in OSCC cells. To test this hypothesis, ChIP‐qPCR against H3K27ac was used. H3K27ac antibody greatly enriched LINC00941 promoter DNA than IgG control. Although the H3K27ac antibody also pulled down LINC00941 promoter from HOK cells, the amount of LINC00941 promoter DNA precipitated from OSCC cells was significantly higher than HOK cells (Figure 2B). To test whether H3K27ac modification affects LINC00941 expression, H3K27ac inhibitor JQ1 was used. As shown, low dose (10 nmol/L) of JQ1 treatment greatly decreased HSC‐3 and OSC‐19 cell LINC00941 promoter H3K27ac modification and LINC00941 gene expression, while no effect was observed on HOK with low dose (10 nmol/L) of JQ1. However, higher dose of JQ1 treatment down‐regulated LINC00941 promoter H3K27ac modification and LINC00941 gene expression across all three cell lines (Figure 3C,D). In summary, these results suggest that high H3K27ac modification in LINC00941 promoter drives LINC00941 expression in OSCC cells. LINC00941 promoter H3K27ac in OSCC is much easier to perturb than normal cells.

### 3.3 EP300 promotes LINC00941 promoter H3K27 acetylation and LINC00941 expression in OSCC cells

EP300 is a histone acetyltransferase that activates transcription through interacting with transcription factors.^25^ We next ask whether LINC00941 promoter H3K27ac modification was mediated through EP300. The enrichment of EP300 on LINC00941 promoter was first tested. Similar to H3K27ac, EP300 signal on LINC00941 promoter was also enriched in HSC‐3 and OSC‐19 cells. However, no statistical difference was observed between IgG and EP300 in HOK cells (Figure 3A). To further understand whether EP300 drives LINC00941 promoter H3K27ac formation and LINC00941 expression. CRISPR‐CAS9 was used to delete EP300 from HOK, HSC‐3 and OSC‐19 cells. The H3K27ac signal on LINC00941 promoter and LINC00941 expression was tested by ChIP‐qPCR and RT‐qPCR, respectively. As indicated, the intensity of H3K27ac signal on LINC00941 promoter and LINC00941 expression was greatly impaired after deleting EP300 in HSC‐3 and OSC‐19 cells (Figure 3B‐D). However, no effect was observed on LINC00941 promoter and LINC00941 expression after deleting EP300 in HOK cells. In addition, when HSC‐3 and OSC‐19 cells were treated with EP300 inhibitor GNE‐049, LINC00941 transcription (Figure 3E) and promoter activity (Figure 3F) were also decreased. EP300 HAT domain is essential for its histone acetyltransferase activity, and we next ask whether EP300 HAT domain is responsible for activating LINC00941 expression. Wild‐type EP300, EP300 mutant in which HAT domain was deleted (EP300dHAT), was exogenously introduced into HSC‐3 and OSC‐19 cells. As expected, EP300 greatly up‐regulated LINC00941 expression; however, EP300dHAT failed to activate LINC00941 (Figure 3G,H). In summary, these results suggest that through its HAT domain, EP300 can selectively promote LINC00941 promoter H3K27 acetylation and LINC00941 expression in OSCC cells.

### 3.4 LINC00941 promotes OSCC cell proliferation and in vivo tumour formation

**FIGURE 4:**
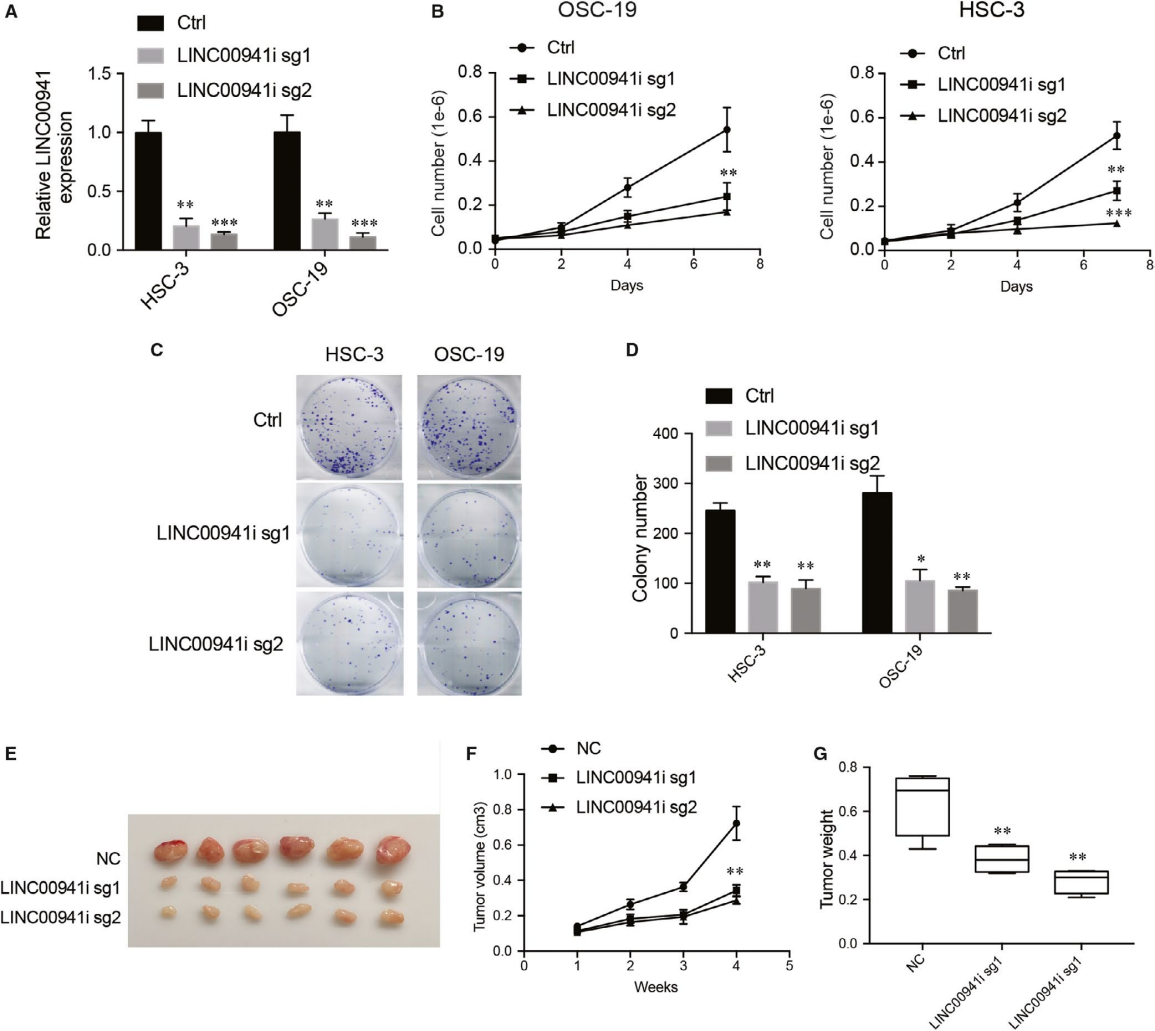
LINC00941 promotes OSCC cell proliferation and in vivo tumour formation. A, Detection of LINC00941 expression after silencing with CRISPR‐dcas9. B, Cell growth after silencing LINC00941. C, Cell colony formation after silencing LINC00941. D, Statistical results of colony number after silencing LINC00941. Mice were killed, and tumour picture was taken 4 wk after starting record tumour growth (E), tumour volume (F) and (G) tumour weight after injecting LINC00941 knockout or wild‐type HSC‐3 cells into nude mice (n = 6) *P < .05, **P < .01, ***P < .001

The role of LINC00941 in OSCC was also examined. Dcas9 tagged with KRAB‐MeCP2 was used to silence LINC00941 expression in OSCC cells. As indicated, both of the sgRNAs used efficiently suppressed LINC00941 expression in HSC‐3 and OSC‐19 cells (Figure 4A). The effect of LINC00941 down‐regulation on OSCC cell growth, colony formation and cell cycle progression was then evaluated. Silencing LINC00941 significantly decreased HSC‐3 and OSC‐19 cell growth and colony formation, and cell cycle was also ceased in G1 phase (Figure 4B‐D). To further understand whether LINC00941 also plays a role in tumour formation. HSC‐3 cells stably expressing dcas9‐KRAB‐MeCP2 were transfected with scramble control or sgRNAs targeting LINC00941 and were injected into nude mice, and tumour formation was then monitored. As expected, suppressing LINC00941 expression also significantly decreased HSC‐3 cell tumour formation (Figure 4E‐G). Thus, these results strongly suggest that LINC00941 plays a critical role in OSCC development.

### 3.5 LINC00941 regulates CAPRIN2 expression through DNA looping

**FIGURE 5:**
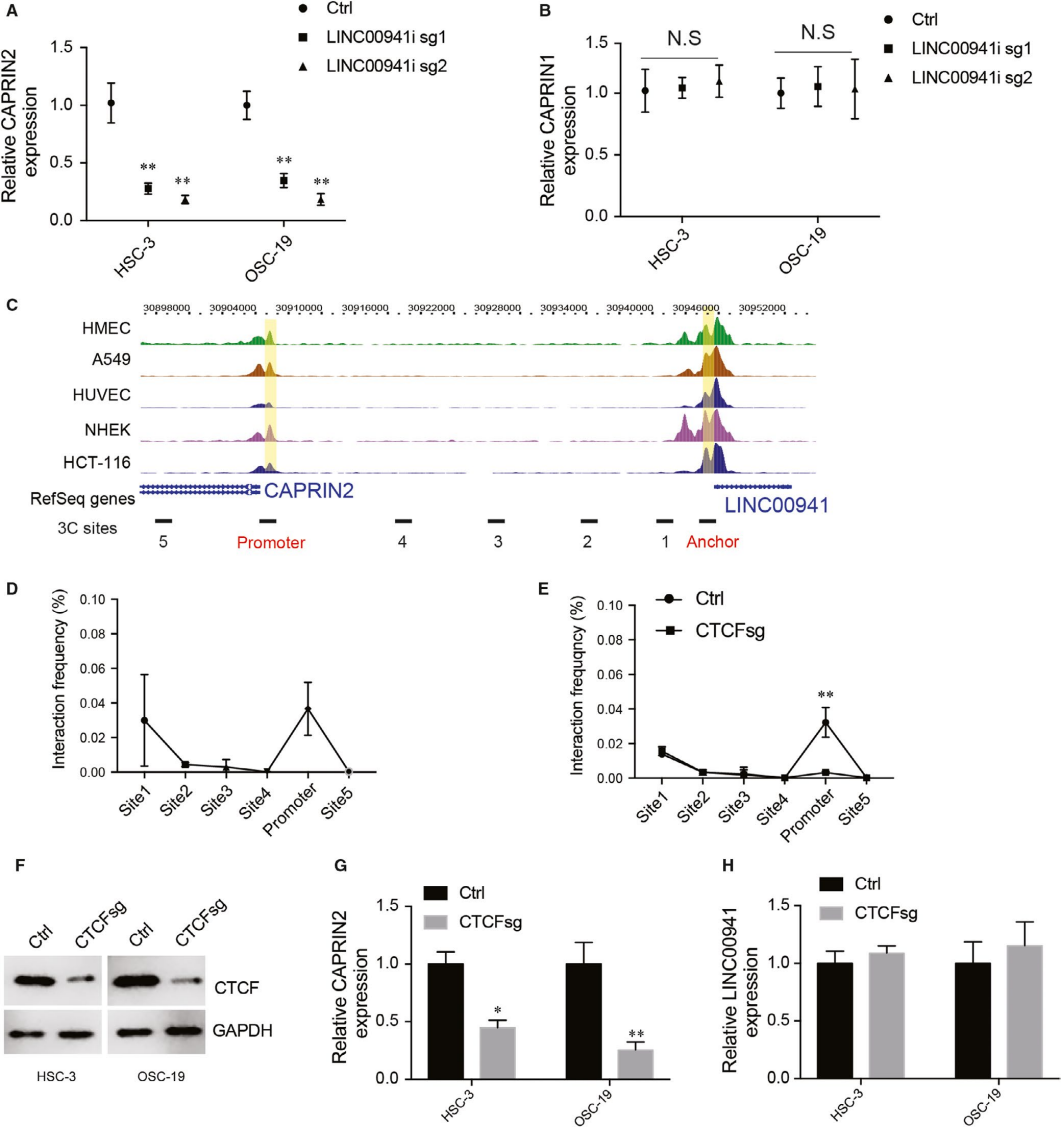
LINC00941 regulates CAPRIN2 expression through DNA looping. A, CAPRIN2 expression after silencing LINC00941. B, CAPRIN1 expression after silencing LINC00941. C, Diagram of H3K27ac signals on LINC00941 and CAPRIN2 across different cancer types. LINC00941 promoter and CAPRIN2 promoter were labelled with yellow. Sites used for DNA‐DNA interaction detection were shown in the bottom. D, 3C results of DNA interaction frequency between anchor and other DNA sites. E, 3C results of DNA interaction frequency between anchor and other DNA sites before and after knocking down CTCF. F, Western blot results of CTCF. GAPDH was used as control. Relative CAPRIN2 (G) and LINC00941 expression (H) after deleting CTCF. *P < .05, **P < .01, ***P < .001

Previous studies showed that long non‐coding RNA regulates gene expression through looping.^26^ CTCF regulates DNA looping and mediates 3D structure of chromatin.^27^ Our motif analysis showed that many CTCF binding sites were enriched within and between LINC00941 and its nearby gene CAPRIN2, and implicating LINC00941 may affect CAPRIN2 expression through looping. To test this hypothesis, CRISPRi was used to silence LINC00941 expression, and CAPRIN2 transcription was then examined by RT‐qPCR. Interestingly, silencing LINC00941 greatly decreased CAPRIN2 expression without affecting CAPRIN1 expression (Figure 5A,B), suggesting LINC00941 can specifically regulate CAPRIN2 expression. To understand whether LINC00941 regulates CAPRIN2 express through DNA looping, 3C was performed in HSC‐3 cells. Primers at LINC00941 promoter were used as an anchor (Figure 5C), and the interactions between the anchor and other selected sites were tested by 3C‐qPCR. The interaction intensity between anchor and other sites was abruptly decreased by distance; however, a strong interaction was detected between the anchor and CAPRIN2 promoter (Figure 5D), suggesting LINC00941 loops to CAPRIN2. To further determine whether CTCF mediates LINC00941 loops to CAPRIN2. CTCF was deleted by CRISPR‐CAS9, and its effect on LINC00941‐CAPRIN2 interaction was tested by 3C‐qPCR. Indeed, the deletion of CTCF almost completely abolished LINC00941 looping to CAPRIN2 (Figure 5E). The regulation of LINC00941 on CAPRIN2 was also impaired as indicated by decreased CAPRIN2 expression after CTCF depletion (Figure 5F,G). No effect was observed on LINC00941 expression after knocking down CTCF (Figure 5H), suggesting LINC00941 regulates CAPRIN2 expression, not vice versa.

### 3.6 CAPRIN2 up‐regulation promotes OSCC cell proliferation

**FIGURE 6:**
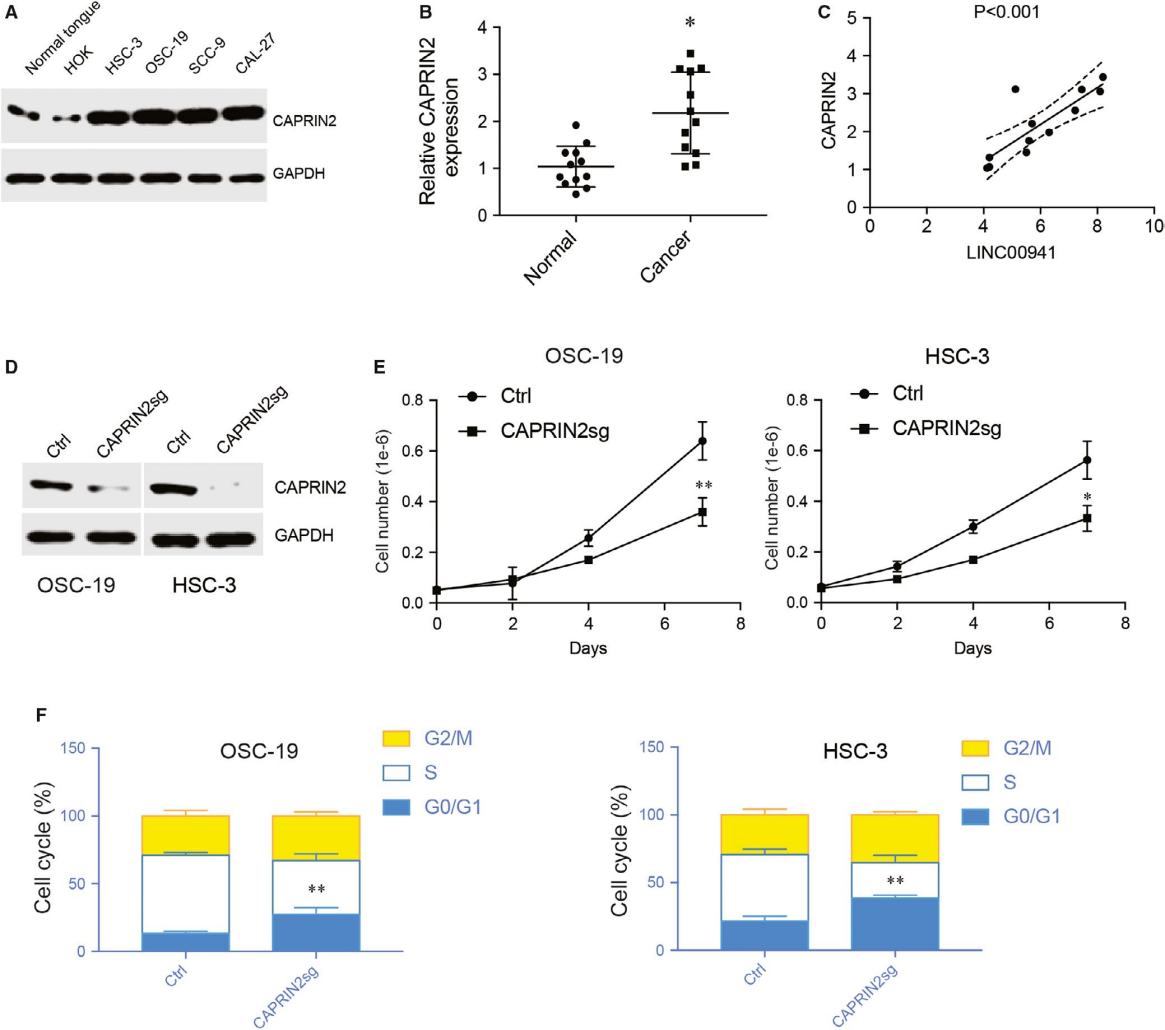
CAPRIN2 up‐regulation promotes OSCC cells proliferation. A, Western blot results of CAPRIN2 expression from OSCC cells, normal oral keratinocytes HOK and normal tongue tissue. B, RT‐qPCR results of CAPRIN2 between patient tumour tissues and normal tissues. C, Correlation analysis of LINC00941 and CAPRIN2 expression between patient tumour tissues and normal tissues. D, Western blot results of CAPRIN2. GAPDH was used as control. E, Cell growth after deleting CAPRIN2. F, Cell cycle detection after deleting CAPRIN2. *P < .05, **P < .01, ***P < .001

LINC00941 up‐regulates CAPRIN2 expression. The expression of CAPRIN2 in OSCC cells and patient samples was examined. Western blot results showed that in comparison with primary tongue tissues and normal oral keratinocytes, CAPRIN2 was expressed at a higher level in OSCC cells (Figure 6A). RT‐qPCR showed that CAPRIN2 was also significantly up‐regulated in tumour tissues than its paired normal tissues (Figure 6B). Correlation analysis demonstrated that LINC00941 was positively correlated with CAPRIN2 expression in patient tumour tissues (Figure 6C). These results suggest that the up‐regulation of LINC00941 also leads to a higher level of CAPRIN2 in OSCC.

We next ask whether CAPRIN2 plays a role in OSCC. CRISPR‐CAS9 was used to delete CAPRIN2 in HSC‐3 and OSC‐19 cells. CAS9 efficiently depleted CAPRIN2 from HSC‐3 and OSC‐19 cells (Figure 6D). HSC‐3 and OSC‐19 cell growth and cell cycle progression were also heavily impaired in the absence of CAPRIN2 (Figure 6E,F), suggesting CAPRIN2 is an essential gene for OSCC.

### 3.7 LINC00941/CAPRIN2 activates canonical WNT signaling pathway

**FIGURE 7:**
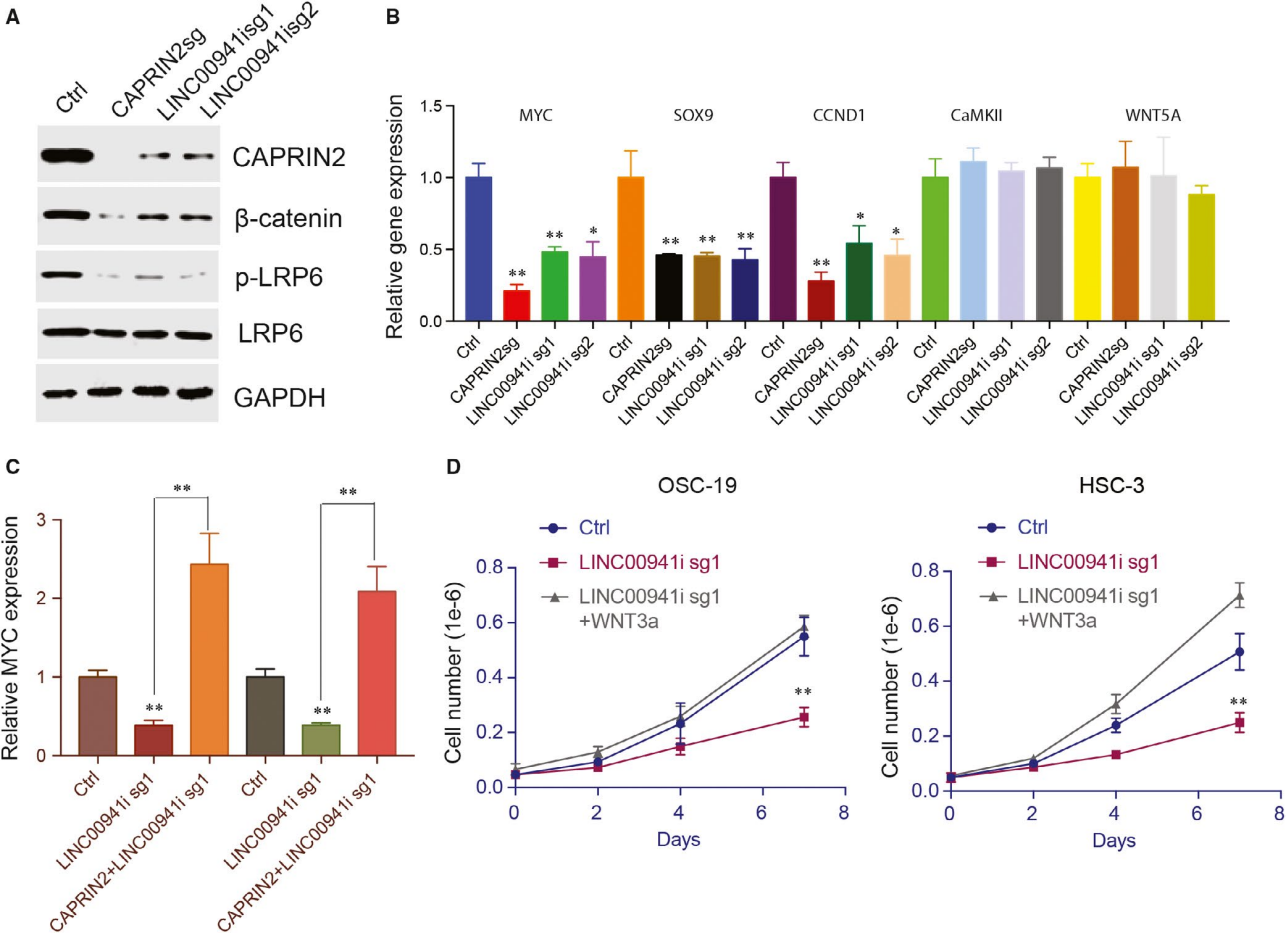
LINC00941/CAPRIN2 activates canonical WNT signaling pathway. A, Western blot results CAPRIN2, β‐catenin, phosphorylated LRP6 (p‐LRP6) and LRP6 after deleting CAPRIN2 or silencing LINC00941. GAPDH was used as control. B, RT‐qPCR detects WNT canonical downstream target genes MYC, SOX9 and CCND1 and WNT non‐canonical downstream gene CaMKII and WNT5a expression after deleting CAPRIN2 or silencing LINC00941. C, MYC expression after silencing LINC00941 in the presence or absence of CAPRIN2. D, Detection of cell growth after silencing LINC00941 in the presence or absence of 30 ng/mL WNT3a. *P < .05, **P < .01, ***P < .001

CAPRIN2 phosphorylates WNT coreceptor LRP6, leading to increased activity of the canonical WNT signaling pathway.^28^ We hypothesis that LINC00941 up‐regulates CAPRIN2 which further promotes WNT signaling pathway activation. To validate this hypothesis, dcas9 was used to silence LINC00941, CAPRIN2 was deleted by CAS9 in HSC‐3 cells, and the expression of genes involved in WNT signaling was then examined. Indeed, knocking down CAPRIN2 or silencing LINC00941 greatly down‐regulated β‐catenin and phosphorylated LRP6 protein level (Figure 7A). The effect of LINC00941 and CAPRIN2 depletion on the non‐canonical WNT signaling pathway was also investigated. As expected, knock‐down of LINC00941 or CAPRIN2 significantly impaired the canonical WNT signaling pathway downstream gene MYC, CCND1 and SOX9 expression; however, genes involved in non‐canonical WNT signaling pathway including WNT5a and CaMKII remain unaffected (Figure 7B). These results suggested that LINC00941 and CAPRIN2 only regulate the canonical WNT signaling pathway in OSCC. We next asked whether CAPRIN2 could rescue the WNT signaling pathway impaired by LINC00941 depletion. CAPRIN2 was introduced into HSC‐3 cells after silencing LINC00941. The impaired MYC expression induced by LINC00941 suppression was completely restored in the presence of CAPRIN2 (Figure 7C). To further understand whether LINC00941 regulates OSCC cell growth through the WNT signaling pathway, WNT3 was used to activate WNT signaling pathway. As shown, cell growth impaired by LINC00941 depletion was greatly rescued after activating the WNT signaling pathway. In all, these results demonstrate that up‐regulation of LINC00941 activates the canonical WNT signaling pathway through CAPRIN2, which further promotes OSCC cell proliferation.

## 4 DISCUSSION

Oral cancer is the sixth most common malignancy in the world.^29^ Despite great advances have been made in the early diagnosis and treatment, the 5‐year survival rate remains at a dismal ~50% in the past few decades.^30^ Improved understanding of the molecular and genetic disorders of this disease is critical for early diagnosis and appropriate treatment. In the present study, we demonstrated that LINC00941 promoter H3K27ac modification mediated by EP300 elevated LINC00941 expression in oral cancer cells. The up‐regulation of LINC00941 further increased CAPRIN2 expression through DNA looping. Moreover, the oncogenic role of LINC00941 in OSCC mediated through CAPRIN2/WNT/ β‐catenin was also revealed.

Emerging evidence suggests that epigenetic alterations, particularly histone modifications and DNA methylation, play a crucial role in the development and progression of human cancer.^31^, ^32^ Histone has a flexible long N‐terminal tail, and lysine residues from its N‐terminal protruding from the histone core nucleosome are susceptible to be modified. Acetylation of Histone 3 at the 27th lysine (H3K27ac) is associated with active transcription and therefore defined as active enhancer marker.^33^ Dysregulation of H3K27ac has been implicated in inappropriate gene regulation and linked to cancer progression.^34^, ^35^ In gastric cancer, the gain of H3K4me3 and H3K27 acetylation on HOXC‐AS3 promoter activated HOXC‐AS3 expression and correlates with poor prognosis.^34^ Aberrant H3K27ac on PLAC2 up‐regulates its transcription which further promotes OSCC cell proliferation through activating WNT3a/β‐catenin signaling pathway.^36^ In this study, through ChIP‐qPCR, we demonstrated that H3K27ac was significantly enriched in OSCC cells in comparison with normal oral keratinocyte cells. Decreasing H3K27ac signals by inhibitor JQ1 greatly impaired LINC00941 promoter H3K27ac signals and LINC00941 expression in OSCC cells. However, at lower doses, the effect of JQ1 on LINC00941 promoter H3K27ac signals and LINC00941 expression in normal oral keratinocyte cells remains intact. These results suggested that JQ1 might be a potential inhibitor for OSCC treatment, although more study should be performed.

EP300 also known as p300 HAT or E1A‐associated protein p300 is a histone acetyltransferase that regulates transcription via chromatin remoulding. EP300 can acetylate all four histones in nucleosomes. It can also activate cAMP signaling pathway by interacting with phosphorylated CREB. Acetylation of H3K27 and H3K122 activates gene transcription. Besides, EP300 can also acetylate non‐histone proteins, such as HDAC1, PRMT1 and ALX1.^37^, ^38^, ^39^ In this study, we found that EP300 can specifically bind to LINC00941 promoter in OSCC cells. Deleting EP300 from OSCC cells greatly impaired LINC00941 promoter H3K27ac modification and LINC00941 expression. Moreover, when OSCC cells were treated with EP300 inhibitor GNE‐049, LINC00941 expression and LINC00941 promoter activity were also greatly inhibited. EP300 acetyltransferase activity was dependent on its HAT domain. Deletion of HAT domain almost completely abolished its transactivity on LINC00941. In all, in this study, we demonstrated that, in OSCC, EP300 mediates LINC00941 promoter H3K27ac modification. EP300 regulates LINC00941 activity largely dependent on its HAT domain.

lncRNA regulates gene expression through several different mechanisms, including functioning themselves as coregulators, regulating basal transcription factor activity, epigenetic modification and post‐transcriptional regulation. Many reports showed that through recruiting transcriptional machinery, lncRNA can regulate its adjacent protein‐coding gene expression. In this study, we found that, through DNA looping, LINC00941 up‐regulates its nearby gene CAPRIN2 expression. Down‐regulation of LINC00941 through CRISPR‐dcas9 impaired CAPRIN2 expression. CTCF is a looping factor that mediates DNA looping and chromatin organization. In this study, we found that depletion of CTCF destroyed LINC00941 loops to CAPRIN2 and down‐regulated CAPRIN2 expression, suggesting LINC00941 loops to CAPRIN2 was dependent on CTCF. In the future, the CTCF binding sites that mediated CTCF dimerization between LINC00941 and CAPRIN2 and which transcription factor is responsible for CAPRIN2 activation should be further studied.

Previous studies showed that, in gastric cancer, aberrant expression of LINC00941 was associated with tumour depth and distant metastasis. Silencing of LINC00941 greatly inhibited gastric cancer cell proliferation, migration and invasion in vitro and modulates tumour growth in vivo.^40^ In contrast, in lung adenocarcinoma, Li et al discovered that high LINC00941 expression was associated with decreased lung adenocarcinoma patient survival.^41^ These seemingly contradictory results suggest that the specific role of LINC00941 in cancer may depend on different contexts. In this study, we found that similar to gastric cancer, high LINC00941 expression was linked to high OSCC cell proliferation, colony formation and tumour growth in vivo. Silencing LINC00941 expression significantly suppressed OSCC cell growth in vitro and tumour formation in vivo. Furthermore, we also demonstrated that in line with LINC00941, CAPRIN2 expression was also up‐regulated and its expression was associated with OSCC cell growth.

Dysregulation of the WNT signaling pathway in oral cancer has been reported through several different molecular mechanisms. For example, Zehang et al discovered that PPP2R5A/WNT signaling pathway can be activated by microRNA‐218, which further promotes cisplatin resistance in oral cancer.^42^ Fubo et al showed that long non‐coding RNA PLAC2 promotes OSCC cell proliferation and invasion through activating WNT/β‐catenin signaling pathway.^36^ TUG1 was reported to promote OSCC progression by activating Wnt/β‐catenin signaling.^43^ In contrast, in OSCC, decreased expression of MEG3 suppressed Wnt/β‐catenin signaling.^44^, ^45^ In this study, we uncovered a new mechanism through which canonical WNT/β‐catenin was activated in OSCC. LINC00941 up‐regulates CAPRIN2 which further activates the canonical WNT signaling pathway and its downstream target gene expression. Overexpression of CPRRIN2 rescued WNT signaling pathway impaired by LINC00941 depletion. In addition, activation of WNT signaling pathway by ligand stimulation also restored OSCC cell growth impaired by LINC00941 suppression. These results support the conclusion that LINC00941 promotes OSCC progression through up‐regulating the CAPRIN2/WNT/β‐catenin signaling pathway.

## CONFLICT OF INTEREST

The authors declare that they have no competing interests.

## AUTHOR CONTRIBUTION

**Haigang Wei:** Data curation (lead); Investigation (lead); Resources (lead); Supervision (lead). **Yilong Ai:** Conceptualization (equal); Methodology (lead); Software (lead). **Siyuan Wu:** Data curation (equal); Resources (equal); Writing‐review & editing (lead). **Chen Zou:** Data curation (equal); Methodology (equal); Validation (equal).

## Data Availability

Data supporting this study are available within this article, and upon requesting with corresponding authors.
